# Chemical Profiling and Biological Screening of Some River Nile Derived-Microorganisms

**DOI:** 10.3389/fmicb.2019.00787

**Published:** 2019-04-12

**Authors:** Momen M. Lotfy, Hossam M. Hassan, Rabab Mohammed, Mona Hetta, Ahmed O. El-Gendy, Mostafa E. Rateb, Mohamed A. Zaki, Noha M. Gamaleldin

**Affiliations:** ^1^Department of Pharmacognosy, Faculty of Pharmacy, Beni-Suef University, Beni-Suef, Egypt; ^2^Department of Pharmacognosy, Faculty of Pharmacy, Nahda University, Beni-Suef, Egypt; ^3^Department of Pharmacognosy, Faculty of Pharmacy, Fayoum University, Fayoum, Egypt; ^4^Department of Microbiology and Immunology, Faculty of Pharmacy, Beni- Suef University, Beni-Suef, Egypt; ^5^School of Computing, Engineering and Physical Sciences, University of the West of Scotland, Paisley, United Kingdom; ^6^Department of Microbiology, Faculty of Pharmacy, The British University in Egypt (BUE), El-Sherouk, Egypt; ^7^The Center for Drug Research and Development (CDRD), The British University in Egypt (BUE), El-Sherouk, Egypt

**Keywords:** chemical profiling, antimicrobial, antileishmanial, antitrypanosomal, antimalarial

## Abstract

**Aims:**

Chemical and biological studies of the River Nile derived-microorganisms are limited. Hence, this work was carried out to screen the River Nile habitat. Identification of the isolated organisms, chemical profiling of their ethyl acetate extracts as well as screening of their antimicrobial, antileishmanial, antitrypanosomal, and antimalarial activities were investigated.

**Methods:**

Identification of the microbial isolates were carried out using bacterial 16S rRNA and fungal 18S rRNA gene sequencing. Chemical profiling of the EtOAc extracts using LC-HRESIMS spectroscopy was carried out. The *in vitro* antimicrobial screening using the modified version of the CLSI method, antileishmanial and antitrypanosomal activities were screened using *Leishmania donovani* promastigote assay, *L. donovani* axenic amastigote assay, *Trypanosoma brucei* trypamastigotes assay and THP1 toxicity assay. The *in vitro* antimalarial activities against D6 (chloroquine sensitive) and W2 (chloroquine-resistant) strains of *Plasmodium falciparum* were evaluated.

**Results:**

Seven isolated microorganisms were identified as *Streptomyces indiaensis, Bacillus safensis, B. anthracis, Bacillus* sp., and *Aspergillus awamori*. Chemical investigation of different extracts showed several bioactive compounds, identified as; nigragillin, 5-caboxybenzofuran and dyramide B from *A. awamori* and actinopolysporin B from *S. indiaensis*. On the other hand many nitrogenous compounds with high molecular weights showed no hits that may correspond to new long chain and/or cyclic peptides. The EtOAc extract of *B. safensis* fermentation broth showed the highest activity against *P. falciparum* D6 and *P. falciparum* W2 (IC_50_ = 25.94 and 27.28 μg/mL, respectively), while two isolates *S. indiaensis* and *Bacillus* sp. RN-011 extracts showed the highest antitrypanosomal activity (IC_50_ = 0.8 and 0.96 μg/mL).

**Conclusion:**

The River Nile could be a new source for production of promising bioactive leading compound where antimicrobial and antiparasitic activities may be correlated.

## Introduction

Nature is an exceptional resource of new biologically active compounds with an astonishing chemical diversity such as that found in plant species, animals, and microorganisms (Butler et al., 2014). Microbial diversity constitutes an infinite pool of novel chemistry, making up a valuable source for innovative biotechnology (Bérdy, 2005; Fenical and Jensen, 2006).

The aquatic environment is now becoming more appreciated as a rich reservoir of new natural products. Ghana and other sub-Saharan African countries are considered as a source of a diverse array of aquatic habitats (Adelaide et al., 2012). In 2012, antibiotic-producing microorganisms were isolated from the Bosomtwe Lake, River Wiwi at KNUST campus and the Gulf of Guinea at Duakor Sea beach. About 27 isolates, out of the 119 recovered, have produced metabolites with antibacterial activity against at least one of the test organisms. The crude extract of the isolate MAI2 (a strain of *Pseudomonas aeruginosa*) was active against all tested organisms; *Bacillus thuringiensis, Proteus vulgaris, Enterococcus faecalis, Staphylococcus aureus, B. subtilis, Escherichia coli, S. typhi*, and *Candida albicans* with MICs ranging between 250 and 2000 μg/mL (Adelaide et al., 2012). A total of 230 Actinomycetes were recovered from the soil of the three-river headwater national nature reserve in Qinghai Province, China. After primary screening, *Streptomyces* sp. SJY056 exhibited strong antagonistic activity against MRSA (Hongjian et al., 2013).

The River Nile is the world’s longest river (Ibrahim, 1984), it extends to 6,800 kilometers in length (Said, 1981). Exclusively, the River Nile manages to cross the Sahara, the world’s largest desert, and reach the Mediterranean Sea. The orientation of Nile basin is unique among the prime rivers in the world, being runs from south to north, discharging at 31°N. Massive variability in precipitation and run-off is also recognized due to different climatic zones it crosses (Chamberlin, 2009).

Chemical profiling of crude natural extracts is considered a challenging analytical task because of the chemical complexity of these extracts. Depending on the type of study, scientists can select major bioactive constituents or minor significant biomarkers (Wolfender et al., 2010). Sparsomycin is an antibiotic that was produced by *Streptomyces* sp. MAR01 isolated from water samples collected from River Nile, Egypt (Atta et al., 2009). LC-HRESIMS is a highly sensitive and efficient technique in the dereplication of natural products by providing accurate masses and molecular formulae which are valuable information for dereplication, being available in the most databases of natural products and independent on the source, the sample preparation and the measurement conditions (Konishi et al., 2007). LC-HRESIMS overcomes the risk of false positive identification which was considered as the most common danger of LC-MS approach since it has the advantage of supplying some information about substructures of natural products (Bobzin et al., 2000; Konishi et al., 2007).

Recently, we have reported the isolation and antimicrobial screening of thirty-four versatile microorganisms from different samples collected from the River Nile (Lotfy et al., 2018). Out of these thirty-four isolates, seven microorganisms were further selected in this study depending on their primary antimicrobial screening results. Herein, we report the identification of these seven isolates using 16S rRNA and fungal 18S rRNA gene sequencing and LC-HRESIMS analysis of the EtOAc extracts of these isolates. Furthermore, these extracts were evaluated for their antimicrobial, antileishmanial, antitrypanosomal, and antimalarial activities.

## Materials and Methods

### Collection, Isolation, and Purification of Nile Derived Microorganisms

Isolation and purification of Nile-derived microorganisms were performed as reported in (Lotfy et al., 2018). Six water and sediment samples were collected from three different localities (Beni-Suef Government, lat. 278939.122- lon. 3197825,71) using collecting spatula. Samples were taken at a depth of two meters and then collected in pre-sterilized bottles and stored in refrigerator at 4°C till analysis.

Isolation of the different microorganisms were carried out using three media; TSA (tryptone soya agar) (Atlas, 1986), SDA (Sabroud Dextrose Agar) (Atlas, 1986), and ISP4 (International Streptomyces Project med. 4 with agar for actinomycetes) (Waksman and Lechevalier, 1962). TSA is composed of (per liter); casein hydrolysate 17 g, soya peptone 3 g, glucose 2.5 g, NaCl 5g, potassium phosphate 2.5 g, and agar 17g, while SDA is composed of (per liter); glucose 20 g, agar 10 g, and peptone 5 g. ISP is composed of (per liter); agar 20 g, glucose 20 g, soluble starch 10 g, CaCO_3_ 2 g, (NH)_4_SO_4_ 2g, K_2_HPO_4_ 1 g, MgSO_4_.7H_2_O 1 g, NaCl 1 g and trace salt solution 1 mL (ferrous sulfate, manganese chloride, copper sulfate, and zinc sulfate 0.01 mg). Pure isolates were obtained using, TSA media, that was supplemented with 50 mg/L nystatin, while SDA was supplemented with 50 mg/L chloramphenicol, and ISP4 was supplemented with 20 mg/L rifampicin and 50 mg/L nystatin during the isolation steps.

The isolation media were autoclaved for 21 min at 120°C, then dispensed in sterilized Petri dishes (9 cm in diameter) and left for solidification. Serial dilutions were made for sediment samples only (water samples were taken without dilution) to cover the range of 10^-1^ to 10^-4^. Screening was carried out by spreading 0.1 mL of each dilution on the surface of the plate containing the isolation agar medium using a sterile glass spreader under aseptic condition. Triplicate plates were prepared for each dilution, and then the plates were left in incubator for 10 days at 30°C. The most suitable dilutions for counting were selected and colonies of bacteria, fungi, and actinomycetes were isolated and purified by re-culturing several times on the same agar medium. The bacterial and fungal isolates were selected according to differences in cultural characteristics.

### Genomic DNA Extraction and Purification

Genomic DNA extraction was done according to (Sinha et al., 2004; Dundar et al., 2015) with some modifications. Briefly, a 1.5 mL of fresh culture was centrifuged for 10 min at 3,000 *g*, the supernatant was discarded, and the pellets were resuspended in 200 μL spheroblast buffer (10% sucrose, 25 mM Tris pH 8.4, 25 mM EDTA pH 8.0, 2 mg/mL lysozyme and 0.4 mg/mL RNase A), vortexed and incubated at 37°C for 10 min until cell lysis occurred. Then, 50 μL of 5% SDS (lysis buffer 1) and 5 M NaCl (lysis buffer 2) were added, mixed and incubated at 65°C for 5 min. A 100 μL neutralizing buffer (60 mL 5 M potassium acetate, 11.5 mL glacial acetic acid, and 28.5 mL dH_2_O) was then added and put on ice for 5 min before centrifugation at 18,000 *g* at 4°C for 15 min. The supernatant (approximately 400 μL) was transferred to a new tube, mixed with equal volume of isopropanol, left for 5 min at 25°C and centrifuged at 18,000 *g* at 25°C for 15 min to precipitate the DNA. The resulting pellet was washed with 70% ethanol by centrifugation at 18,000 *g* at room temperature for 5 min. The final pellet was air-dried and re-suspended in 50 μL 1 × TE buffer pH 8 and stored in the refrigerator at 4°C.

### PCR Amplification and Sequencing of Bacterial 16S rRNA and Fungal 18S rRNA Genes

PCR was carried out in 50 μL reaction volume in a sterile 200 μL PCR tube. The PCR reaction mixture consisted of 500 ng genomic DNA, 10 mM dNTPs mixture, 1 μL (20 uM of each primer), 2.5 units of Taq DNA polymerase enzyme and 10 μL 5X reaction buffer. The PCR program included template denaturation at 94°C (3 min), followed by 34 cycles of denaturing at 94°C (30 s), annealing at 56°C (30 s), and extension at 72°C (60 s), and followed by completion of DNA synthesis at 72°C (5 min). Primers were removed from the final PCR product before sequencing using QIAquick PCR purification kit (QIAGEN, Germany). The PCR product of interest was detected and purified by agarose gel electrophoresis using 1% (w/v) agarose gels with reference to 1 kbp DNA ladder. DNA was sequenced using the ABI Prism BigDye terminator sequencing ready reaction kit version 3.1 and analyzed with the ABI Prism 3100 generic analyzer.

### Sequence Manipulation and Phylogenetic Analysis

The BLAST facility^1^ was employed to assess the degree of DNA similarity. Multiple sequence alignment and molecular phylogeny were evaluated using MEGA7 software (Tamura et al., 2007).

### Preparation of River Nile-Derived Microbial Extracts

Pure cultures of the isolated microorganisms were incubated in 150 mL liquid medium that used in purification step (Lotfy et al., 2018) and kept for 7–10 days under continuous shaking (150 rpm) at 30°C. The culture broth for each isolate was then extracted by EtOAc, and then the solvent was evaporated. Each solvent-free residue was divided into two portions. One of them was subjected to LC-HRESIMS analysis while the other portion was kept for biological study.

### LC-HRESIMS Profiling of the Metabolites Produced by Different Isolated Microorganisms

LC-HRESIMS spectrometric technique is composed of Thermo Instruments MS system (LTQ XL/LTQ Orbitrap Discovery) coupled to a Thermo Instruments HPLC system (Accela PDA detector, Accela PDA autosampler and Accela Pump). The following conditions were used: capillary voltage 45 V, capillary temperature 260°C, and auxiliary gas flow rate 10–20 arbitrary units, sheath gas flow rate 40–50 arbitrary units, spray voltage 4.5 kV, mass range 100–2000 amu (maximum resolution 30000). For LC/MS; Waters SunFire C_18_ RP analytical HPLC column (5 μm, 4.6 × 150 mm) using a gradient of MeOH in H_2_O containing 0.01% formic acid as eluent (0–100% over 30 min) at a flow rate 1 mL/min. (Marine Biodiscovery Centre, Chemistry Department, University of Aberdeen).

### Biological Study

#### Antimicrobial Effect of the Crude Extracts

The crude extracts were prepared as a stock solution of 2 mg/mL in DMSO then diluted four folds with incomplete RPMI medium to 0.5 mg/mL.

All organisms were obtained from the American Type Culture Collection (Manassas, VA, United States) and included the fungi: *C. albicans* ATCC90028, *C. glabrata* ATCC90030, *C. krusei* ATCC6258, *Cryptococcus neoformans* ATCC90113, and *Aspergillus fumigatus* ATCC204305, and the bacteria: *S. aureus* ATCC29213, *methicillin-resistant S. aureus* ATCC33591 (MRSA), *E. coli* ATCC35218, and *P. aeruginosa* ATCC27853. Susceptibility testing was performed using a modified version of the CLSI (formerly NCCLS) method (Michael et al., 2002; Gail et al., 2003; Matthew et al., 2006). Samples were serially diluted in 20% DMSO/saline and transferred in duplicate to 96-well flat-bottomed microplates. Microbial inocula were prepared by correcting the OD_630_ of microbe suspensions in incubation broth to give final target inocula. Drug controls [Ciprofloxacin (ICN Biomedicals, Solon, OH, United States) for bacteria and Amphotericin B (CN Biomedicals, OH, United States) for fungi] were included in each assay. All organisms were read at either 530 nm using the Biotek Powerwave XS plate reader (Bio-Tek Instruments, Winooski, VT, United States or 544ex/590em, (*A. fumigatus*) using the Polarstar Galaxy Plate Reader (BMG Lab Technologies, Ortenburg, Germany) before and after incubation. Minimum fungicidal or bactericidal concentrations were determined by removing 5 μL from each clear well, followed by transferring to agar and incubating. The MFC/MBC is defined as the lowest test concentration that kills the organism (allows no growth on agar). IC_50_ values were determined from dose-response curves of per cent decrease in cell viability against test concentrations. Ciprofloxacin was used as an antibacterial positive control against *S. aureus*, MRSA, *E. coli, P. aeruginosa* and exhibited IC_50_ value of 0.033, 0.019, 0.003, and 0.019 μg/mL, respectively. Amphotericin B was used as an antifungal positive control against *C. albicans, C. glabrata, C. krusei, A. fumigatus*, and *C. neoformans* and exhibited IC_50_ value of 0.157, 0.203, 0.526, 1.201, and 0.157 μg/mL, respectively. DMSO was used as the negative (vehicle) control.

#### Antiprotozoal Activity

##### *In vitro* antileishmanial and antitrypanosomal assays

Samples with a stock concentration of 2 mg/mL in DMSO were diluted four folds with incomplete RPMI medium to 0.5 mg/mL.

The diluted samples were tested for different activities namely, *Leishmania donovani* promastigote assay, *L. donovani* axenic amastigote assay, *L. donovani* THP1 macrophage amastigote assay, *Trypanosoma brucei* assay and THP1 (Tamm–Horsfall protein) toxicity assay using Alamar Blue assays were used (Rahman et al., 2011; Manda et al., 2014; Jain et al., 2016). These assays were adapted to 384 well micro-plate format. A 3–4 days’ old culture of *L. donovani* promastigotes in the exponential phase was diluted with RPMI medium to 1 × 10^6^ cells/mL for promastigote assays. A 3–4 days’ old culture of *L. donovani* axenic amastigotes was diluted with RPMI medium to 2 × 10^6^ cells/mL for axenic amastigote assays. A 2 days’ old culture of *Trypanasoma brucei* in the exponential phase was diluted with IMDM medium to 5 × 10^3^ cells/mL for the antitrypanosomal assay. The samples with appropriate dilution as mentioned above were added to the *L. donovani* promastigotes or *L. donovani* axenic amastigotes or *T. bruei* trypamastigotes cultures. All samples were tested at three concentrations ranging from 10 to 0.4 μg/mL. The plates were incubated at 26°C for 72 h (37°C for axenic amastigotes and *T. brucei* trypamastigotes) and growth of the parasites in cultures was determined by Alamar Blue assay (Rahman et al., 2011; Manda et al., 2014; Jain et al., 2016).

Samples were also tested against *L. donovani* intracellular amastigotes in THP1 cells employing a recently developed parasite-rescue and transformation (Jain et al., 2012). THP1 toxicity assay and Macrophage Amastigote assay. A 4-day-old THP1 cell culture in the exponential phase was diluted with RPMI medium to 2.5 × 10^5^ cells/ml. PMA was added to final a concentration of 25 ng/mL. PMA treated culture was dispensed in experimental culture plates and incubated overnight at 37°C in a 5% CO_2_ incubator. The plates with differentiated THP1 Cells were washed with serum-free medium. For THP1 toxicity assay, diluted test samples were added over differentiated THP1 cells and plates were incubated for 48 h at 37°C in a 5% CO_2_ incubator. The cell growth was determined by Alamar Blue assay. IC_50_ and IC_90_ values were computed from the dose-response curves using XLFit^®^.

##### *In vitro* antimalarial activity

The *in vitro* antimalarial activity was determined against D6 (chloroquine sensitive) and W2 (chloroquine-resistant) strains of *Plasmodium falciparum*, which were obtained from the Division of Experimental Therapeutics, Walter Reed Army Institute of Research (WRAIR), Washington, DC. The assay is based on the determination of plasmodial lactate dehydrogenase (LDH) activity. A suspension of red blood cells infected with strain D6 or W2 strain of *P. falciparum* (200 μl, with 2% parasitemia and 2% hematocrit in RPMI 1640 medium supplemented with 10% human serum and 60 μg/ml amikacin) was added to the wells of a 96-well plate containing 10 μL of serially diluted test samples. The plate was incubated at 37°C for 72 h in a modular incubation chamber flushed with a gas mixture of 90% N_2_, 5% O_2_, and 5% CO_2_. Parasite LDH activity was determined according to the procedure of Makler and Hinrichs (1993). Twenty microliters of the incubation mixture were mixed with 100 μL of Malstat reagent and incubated at room temperature for 30 min. Twenty microliters of a 1:1 mixture of nitroblue tetrazolium and phenazine ethosulfate (Sigma, St. Louis, MO, United States) was then added, and the plate was further incubated in the dark for 1 h. The reaction was stopped by adding 100 μL of 5% acetic acid. The plate was read at 650 nm. Percent growth was plotted versus test concentrations. IC_50_ were obtained from the dose-response curves. Artemisinin and chloroquine were included as drug controls, and dimethyl sulfoxide was included as a vehicle control. To determine the selectivity index (SI) of the antimalarial activity of test compounds, their *in vitro* cytotoxicity to mammalian cells was also determined. The assay was performed with 96-well tissue culture-treated plates. Vero cells [monkey kidney fibroblasts (American Type Culture Collection, Manassas, VA, United States)] were seeded into the wells of 96-well plates at a density of 25,000 cells/well and incubated for 24 h. Samples at different concentrations are added, and the plates were again incubated for 48 h. The number of viable cells was determined by Neutral Red assay (Borenfreund et al., 1990). IC_50_ were obtained from dose-response curves.

## Results

Thirty-four isolates were obtained pure forms using three different media (TSA, SDA, and ISP4), varying between *Streptomyces* sp., *Bacillus* sp., *Aspergillus* sp., and *Fusarium* sp. Based on physical examination, metabolic characteristics and primary antimicrobial screening that was reported in Lotfy et al. (2018), seven isolates (out of thirty-four isolates) were selected. Small scale-fermented and extracted with EtOAc were analyzed using LC/HRMS. On the other hand the full identification of the selected isolates were carried out using 16S rRNA and 18S rRNA genes sequencing.

### DNA Sequencing for Selected Isolates and Phylogenetic Analysis

**FIGURE 1:**
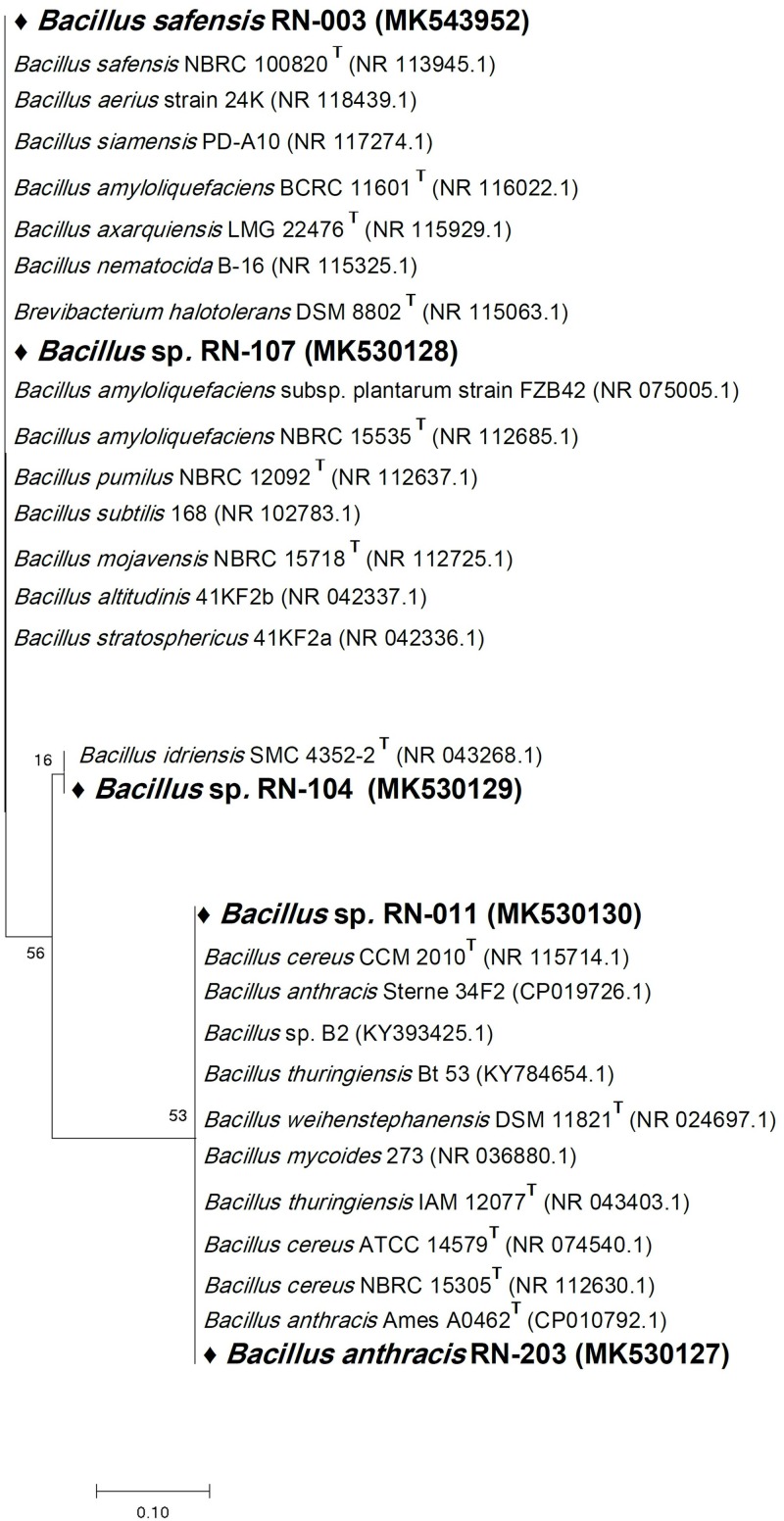
Phylogenetic tree of the River Nile derived isolates based on partial 16S rRNA gene sequences. The phylogenetic tree was inferred using the Neighbor-Joining method (Saitou and Nei, 1987). The distances were computed using the Kimura 2-parameter method (Kimura, 1980) and are in the units of the number of base substitutions per site. Numbers at nodes indicate percentages of 1000 bootstrap re-samplings. All positions containing gaps and missing data were eliminated. Evolutionary analyses were conducted in MEGA7 (Kumar et al., 2016).

**FIGURE 2:**
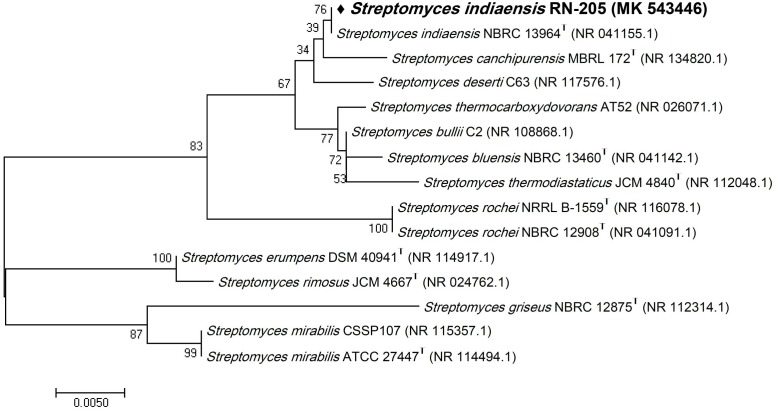
Phylogenetic tree of the River Nile derived *Streptomyces indiaensis* RN-205 based on partial 16S rRNA gene sequences. The phylogenetic tree was inferred using the Neighbor-Joining method (Saitou and Nei, 1987). The distances were computed using the Kimura 2-parameter method (Kimura, 1980) and are in the units of the number of base substitutions per site. Numbers at nodes indicate percentages of 1000 bootstrap re-samplings. All positions containing gaps and missing data were eliminated. Evolutionary analyses were conducted in MEGA7 (Kumar et al., 2016).

**FIGURE 3:**
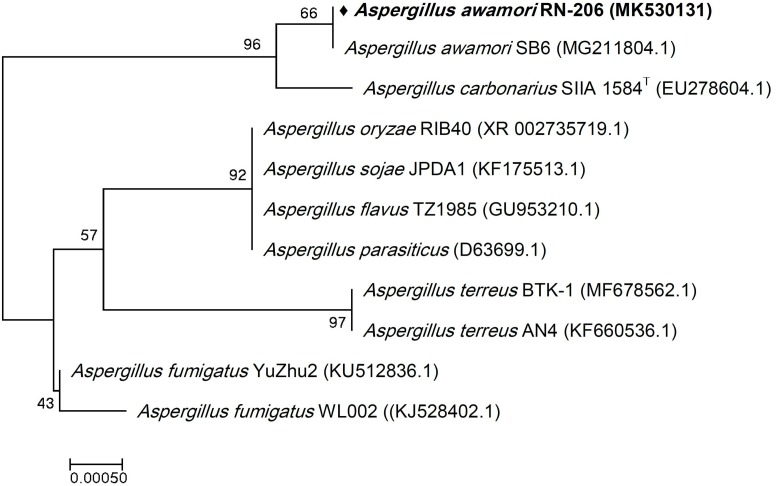
Phylogenetic tree of the River Nile derived *Aspergillus awamori* RN-206 based on partial 18S rRNA gene sequences. The phylogenetic tree was inferred using the Neighbor-Joining method (Saitou and Nei, 1987). The distances were computed using the Kimura 2-parameter method (Kimura, 1980) and are in the units of the number of base substitutions per site. Numbers at nodes indicate percentages of 1000 bootstrap re-samplings. All positions containing gaps and missing data were eliminated. Evolutionary analyses were conducted in MEGA7 (Kumar et al., 2016).

Six bacterial and one fungal DNA samples were extracted. The extracted ultra-pure DNA samples were analyzed (Macrogen Company, South Korea), the results are shown in (Table 1). The resulted sequences were aligned to the closely related microorganisms by retrieving their sequences from the NCBI GenBank database and assembled in MEGA7 software for phylogenetic analysis using the Neighbor-Joining method and the evolutionary distances were computed using the Kimura 2-parameter method (Kimura, 1980) as seen in (Figure 1–3). All sequences for strains RN-203, RN-107, RN-104, RN-011, RN-206, RN-205, and RN-003 were deposited in the GenBank and assigned with accession numbers MK530127, MK530128, MK530129, MK530130, MK530131, MK543446, and MK543952, respectively (Table 1).

**Table 1:** Results for DNA sequencing using NCBI GenBank database.

Strain ID identifier	Own accession number	NCBI BLAST result	Accession number of the closest relative	Max score	Total score	Query cover	*E*-value	Max ident.
RN-205	MK543446	*Streptomyces indiaensis* strain At5 16S ribosomal RNA gene, partial sequence	KP662886.1	719	719	100%	0.0	100%
RN-003	MK543952	*Bacillus safensis* strain NBRC 100820 16S ribosomal RNA gene, partial sequence	NR_113945.1	946	1398	100%	0.0	100%
RN-203	MK530127	*Bacillus anthracis str.* Ames strain Ames 16S ribosomal RNA, complete sequence	NR_074453.1	2713	2713	100%	0.0	100%
RN-206	MK530131	*Aspergillus awamori* strain K-03 18S ribosomal RNA gene, partial sequence	KF922319.1	2922	2922	100%	0.0	100%
RN-011	MK530130	*Bacillus cereus* strain CCM 2010 16S ribosomal RNA gene, complete sequence	NR_115714.1	2507	2507	74%	0.0	97%
RN-104	MK530129	*Bacillus halotolerans* strain RTAE04 16S ribosomal RNA gene, partial sequence	KX530769.1	2222	2222	100%	0.0	98%
RN-107	MK530128	*Bacillus amyloliquefaciens* subsp. plantarum strain FZB42 16S ribosomal RNA gene, complete sequence	NR_075005.1	2612	2612	80%	0.0	98%


### LC-HRESIMS Profiling of the Metabolites Produced by the Isolated Microorganisms

**FIGURE 4:**
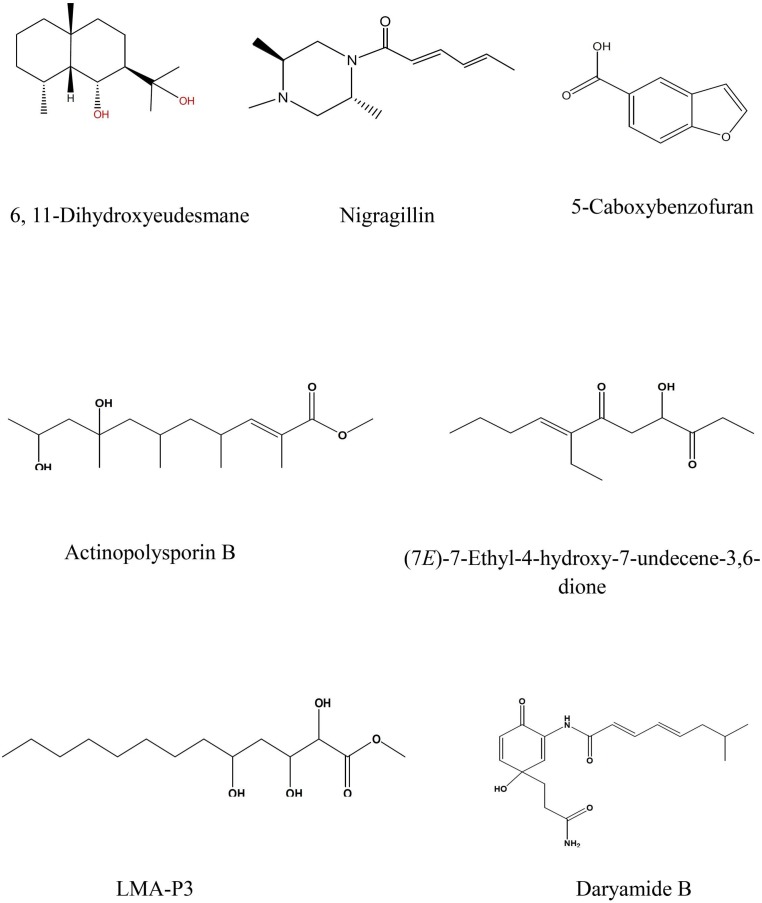
Selected structures of predicted chemical formulas from LC-HRESIMS analysis for the isolated microorganisms.

LC-HRESIMS analysis revealed the presence of several compounds that were determined by comparison with previously isolated compounds with the aid of different libraries databases as shown in (Figure 4 and Table 2).

**Table 2:** Results of LC-HRESIMS analysis for the selected microorganisms EtOAc extracts.

No.	EtOAc extracts of isolated microorganism	Predicted molecular formula	Retention time	Predicted structure
1	RN-206	C_13_H_22_ON_2_	3.96	Nigragillin
2		C_9_H_6_O_3_	7.28	5-Caboxybenzofuran)
3		C_18_H_24_N_2_O_4_	7.92	Daryamide B
4		C_19_H_37_O_6_N	8.77	No hits
5		C_53_H_74_O_12_N_10_	10.95	No hits
6		C_54_ H_76_O_11_N_10_	11.6	No hits
7	RN-203	C_53_H_74_O_12_N_10_	10.99	No hits
8		C_54_H_76_O_12_N_10_	11.8	No hits
9		C_55_H_78_O_12_N_10_	12.59	No hits
10	RN-205	C_13_H_22_O_3_	5.89-6.12	(7*E*)-7-ethyl-4-hydroxy-7-undecene-3,6-dione
11		C_14_H_28_O_5_	7.12	LMA-P3
12		C_16_H_30_O_4_	8.52	Actinopolysporin B
13		C_16_H_32_O_5_	8.52	No hit
14		C_15_H_28_O_2_	10.13	6,11-dihydroxyeudesmane
15	RN-011	C_14_ H_20_ O_2_ N_2_	5.23	No hits
16		C_12_H_20_O_3_	7.00	No hits
17		C_12_H_23_ON	8.18	No hits
18		C_12_H_25_O_2_N	8.65	No hits
19		C_12_H_23_O_2_N	8.93	No hits




The EtOAc extract of *Aspergillus awamori* (RN-206) showed six major peaks; nigragillin, 5-caboxybenzofuran and dyramide B, together with three compounds with molecular formulae C_19_H_37_O_6_N, C_53_H_74_O_12_N_10_, and C_54_H_76_O_12_N_10_ which could be new alkaloid and peptide candidates.

Actinopolysporin B was detected in the EtOAc extract of the culture broth of *Streptomyces indiaensis* (RN-205), together with a compound with the molecular formula C_16_H_32_O_2_ which has no natural hits but could be proposed as a new actinopolysporin B derivative, three known compounds were also found in the same extract; 6,11-dihydroxyeudesmane, (7*E*)-7-ethyl-4-hydroxy-7-undecene-3,6-dione and LMA-P3. On the other hand, the remaining microbial extracts showed no-hit structures in the databases suggesting the presence of new scaffold compounds.

### Biological Study

#### Antimicrobial Activities

The antimicrobial screening was carried on the EtOAc extracts of the selected seven isolates using a modified version of the CLSI (formerly NCCLS) method. EtOAc extracts of the isolated microorganisms showed no significant activity against opportunistic infections as shown in (Table 3).

**Table 3:** Antimicrobial screening of EtOAc extracts of selected isolates using CLSI method (OI).

Sample code	*C. albicans* IC_50_ (μg/ml)	*A. fumigatus* IC_50_ (μg/ml)	*C. neoformans* IC_50_ (μg/ml)	MRSA IC_50_ (μg/ml)	*E. coli* IC_50_ (μg/ml)	*P. aeruginosa* IC_50_ (μg/ml)	*K. pneumonia* IC_50_ (μg/ml)	VRE IC_50_ (μg/ml)
RN-205	>200	>200	>200	>200	>200	>200	>200	>200
RN-206	>20	>20	>20	>20	>20	>20	>20	>20
RN-203	>200	>200	>200	>200	>200	>200	>200	>200
RN-011	>200	>200	>200	>200	>200	>200	>200	>200
RN-107	>200	>200	>200	>200	>200	>200	>200	>200
RN-104	>20	>20	>20	>20	>20	>20	>20	>20
RN-003	>200	>200	>200	>200	>200	>200	>200	>200
Cipro	ND	ND	ND	0.019	0.003	0.019	ND	ND
Amb	0.157	1.201	0.157	ND	ND	ND	ND	ND


*Cipro, Ciprofloxacin; Amb, Amphotericin B; ND, no data.*

#### Antiprotozoal Activity

Antileishmanial activity was carried out using *L*. *donovani* promastigote assay, *L. donovani* axenic amastigote assay, and *L. donovani* THP1 macrophage amastigote assay. Results showed that no significant antileishmanial activity. However, the EtOAc extract of two isolates; RN-205 (*S. indiaensis* At5) and RN-011 (*Bacillus* sp. RN-011) showed high antitrypanosomal activity with IC_50_ = 0.8 and 0.96 μg/mL, respectively, while RN-206 (*A. awamori* K03) showed moderate antitrypanosomal activity (IC_50_ = 3.49 μg/mL) as shown in (Table 4).

**Table 4:** Antileishmanial and antitrypanosomal assays of the EtOAc extracts of the isolated microorganisms showing high antitrypanosomal activity by metabolites of *Streptomyces indiaensis* RN-205 and *Bacillus* sp. RN-011.

Sample Code	*L. donovani* Promastigote		*L. donovani* Amastigote		*L. donovani* Amastigote/THP		*T*. *brucei*		THP1	
					
	IC_50_ (μg/ml)	IC_90_ (μg/ml)	IC_50_ (μg/ml)	IC_90_ (μg/ml)	IC_50_ (μg/ml)	IC_90_ (μg/ml)	IC_50_ (μg/ml)	IC_90_ (μg/ml)	IC_50_ (μg/ml)	IC_90_ (μg/ml)
RN-205	>20	>20	>20	>20	>20	>20	<0.8	1.03	>20	>20
RN-206	>10	>10	>10	>10	>10	>10	3.49	6.39	>10	>10
RN-203	>20	>20	>20	>20	>20	>20	4	7.62	>20	>20
RN-011	>20	>20	>20	>20	>20	>20	0.96	1.23	>20	>20
RN-003	>20	>20	>20	>20	>20	>20	>20	>20	>20	>20
RN-107	>20	>20	>20	>20	>20	>20	1.11	1.66	>20	>20
RN-104	>10	>10	>10	>10	>10	>10	3.52	6.76	>10	>10
Amb	0.165	0.212	0.308	0.361	0.147	0.213	ND	ND	ND	ND
Pm	1.661	2.594	9.946	ND	1.050	5.915	ND	ND	ND	ND
DFMO	ND	ND	ND	ND	ND	ND	3.960	9.159	ND	ND


*Amb, Amphotericin B; Pm, Pentamidine; DFMO, Difluoromethylornithine; ND, no data.*

**Table 5:** Antimalarial assays of the EtOAc extracts of the isolated microorganisms showing that metabolites from *Bacillus safensis* RN-003 exhibited moderate antiplasmodial activity and that from *Bacillus anthracis* RN-203 showed weak activity.

Sample Code	*P. falciparum* D6	*P. falciparum* W2	VERO
			
	IC_50_ (μg/ml)	IC_50_ (μg/ml)	IC_50_ (μg/ml)
RN-205	>47.60	>47.60	>47.60
RN-206	>47.60	>47.60	>47.60
RN-203	45.565	31.624	>47.60
RN-011	>47.60	>47.60	>47.60
RN-107	>47.60	>47.60	>47.60
RN-104	>47.60	>47.60	>47.60
RN-003	25.939	27.278	>47.60


The *in vitro* antimalarial activity was determined against D6 (chloroquine sensitive) and W2 (chloroquine-resistant) strains of *P. falciparum*. Results showed that one isolate RN-003 (*Bacillus safensis* NBRC 100820) exhibited moderate antiplasmodial activity (IC_50_ = 25.94 and 27.28 μg/mL) against *P. falciparum* D6 and *P. falciparum* W2, respectively. Additionally, RN-203 (*B. anthracis* Ames) showed weak activity against *P. falciparum* D6 and *P. falciparum* W2 (IC_50_ = 45.57 and 31.62 μg/ml, respectively) as shown in (Table 5).

## Discussion

The discovery of new natural sources for drug discovery is quite challenging. Investigation of new habitats in microbiologically un- or under-explored habitats offers the advantage of finding novel microorganisms and bioactive metabolites (Penesyan et al., 2010). The River Nile as an unexplored habitat could be a store house for new versatile microbes. Hence, it could be considered as a great and renewable source for the isolation of bioactive metabolites, which constitutes a potential biotechnological stream and overcomes drawbacks of synthetic drugs and the low yield of natural products derived from plants.

Replication, the re-isolation of known compounds, has become a significant challenge in the drug discovery area which consumes time, effort, and money. Thus, dereplication is an essential process in the drug discovery from natural sources. It accelerates the discovery of novel natural products by eliminating repetitive work on known natural products using available databases and sensitive spectroscopic technique like LC-HRESIMS.

LC-HRESIMS analysis of the selected isolates revealed that seven compounds were identified; three compounds were identified from *A. awamori*, while four compounds were identified from *S. indiaensis*. Some of these compounds are known to have a broad spectrum of biological activities including antimicrobial, cytotoxic and antioxidant activities, such as 6,11-dihydroxyeudesmane, a member of eudesmane-type sesquiterpenes family which exhibited a wide range of biological activities including cytotoxic (Bomfim et al., 2013; Zhao et al., 2014), antibacterial (Iken and Baker, 2003; Li et al., 2015) and inhibitory activity against acetyl cholinesterase and nitric oxide production in LPS-induced murine macrophages (Xu et al., 2012; Jiang et al., 2013; Liu et al., 2014), which could also be responsible for the high antitrypanosomal activity of *S. indiaensis* At5 (RN-205).

On the other hand, several compounds which showed no hits with different molecular formulae were detected indicating new/novel bioactive metabolites. This finding recommends a future large scale fermentation, especially for four isolates; *Bacillus* sp. (RN-011), *A. awamori* K03 (RN-206), *B. safensis* NBRC100820 (RN-003), and *B. anthracis* Ames (RN-203) to isolate and identify these hits that may lead to the isolation of new derivatives or lead compounds. These constituents may also provide the scientific evidence for these extracts’ promising antiprotozoal activities.

## Conclusion and Future Aspects

Based on our microbiological, chemical and biological investigations of the River Nile, seven microorganisms were identified using bacterial 16S rRNA and fungal 18S rRNA gene sequencing. Their promising antimicrobial and antiprotozoal activities in addition to LC-HRESIMS analysis of the EtOAc extracts of these isolates recommended large-scale fermentation for four of them to isolate the no-hits which could be new candidates or new derivatives of known compounds with a wide range of biological activities.

In conclusion, the River Nile could be a wonderful source for the isolation of new microbes and the production of bioactive metabolites. So, it was interesting to investigate this valuable source while we believe that it is not only a gift to Egypt because being the source of drinking and irrigating waters, but also it is the mysterious ability for curing.

## Author Contributions

ML and AE-G performed the microbiological and molecular biology experiments. HH and MR worked on LC-HRESIMS spectroscopy and chemical profiling. MZ, RM, MH, and NG performed the antileishmanial, antitrypanosomal, and antimalarial activities. HH, RM, and MH put the study design. ML drafted the manuscript. All authors revised and approved the final manuscript.

## Conflict of Interest Statement

The authors declare that the research was conducted in the absence of any commercial or financial relationships that could be construed as a potential conflict of interest.

## Footnotes

1 www.ncbi.nlm.nih.gov/blst
